# Rare case of concomitant coronary artery bypass grafting and open abdominal aortic aneurysm repair

**DOI:** 10.1093/jscr/rjae672

**Published:** 2024-10-28

**Authors:** Dakota Pastore, Sabrina Higgins, Taylor James, Zamaan Hooda, Pasha Shenasen, Alexios Therionos, John Paul Bustamante, Jagbir Beniwal, Mark Connolly, John Danks

**Affiliations:** Department of Surgery, St. Joseph’s University Medical Center, 703 Main St. Paterson, NJ 07503, United States; Department of Surgery, St. Joseph’s University Medical Center, 703 Main St. Paterson, NJ 07503, United States; Department of Surgery, St. Joseph’s University Medical Center, 703 Main St. Paterson, NJ 07503, United States; Department of Surgery, St. Joseph’s University Medical Center, 703 Main St. Paterson, NJ 07503, United States; Department of Surgery, St. Joseph’s University Medical Center, 703 Main St. Paterson, NJ 07503, United States; Department of Surgery, St. Joseph’s University Medical Center, 703 Main St. Paterson, NJ 07503, United States; Department of Surgery, St. Joseph’s University Medical Center, 703 Main St. Paterson, NJ 07503, United States; Department of Surgery, St. Joseph’s University Medical Center, 703 Main St. Paterson, NJ 07503, United States; Department of Surgery, St. Joseph’s University Medical Center, 703 Main St. Paterson, NJ 07503, United States; Department of Surgery, St. Joseph’s University Medical Center, 703 Main St. Paterson, NJ 07503, United States

**Keywords:** abdominal aortic aneurysm, coronary artery bypass grafting, hypertension, open aortic aneurysm repair, hypertension, coronary artery disease

## Abstract

Coronary artery disease occurs when coronary vessels are unable to supply adequate oxygen to the myocardium, while an abdominal aortic aneurysm (AAA) is a dilatation of the abdominal aorta. Both conditions have similar risk factors such as smoking and hypertension. If these disease processes become severe and are left untreated, life-threatening consequences may occur. We present a 71-year-old male with prior myocardial infarction and an infrarenal AAA that underwent a rare combined procedure of coronary artery bypass grafting (CABG) and open AAA repair surgery. The CABG involved grafting the left internal mammary artery and saphenous vein to coronary arteries while the AAA repair used an 18-mm bifurcated aortic graft. Combined CABG and open AAA repair is complex and rare, but aims to reduce mortality and prevent aneurysm rupture in patients with severe comorbid conditions. The patient’s positive postoperative outcome highlights the procedure’s efficacy in select cases.

## Introduction

Coronary artery bypass grafting (CABG) is a major cardiothoracic surgical procedure to bypass atherosclerotic coronary vessels with the use of conduits grafted from venous or arterial sources to restore myocardial perfusion [1]. Patients found to have high-grade occlusion of main coronary vessels or have failed percutaneous coronary intervention are candidates for CABG [1]. This surgery is considered high risk as it puts patients at risk for various complications such as ischemic stroke, graft rejection, and death [1]. However, this surgery has the potential to decrease mortality from coronary artery disease (CAD) [2].

Abdominal aortic aneurysms (AAAs) are localized dilations of the abdominal aorta due to vessel wall degeneration [3–5]. Any pathology that compromises the internal elastic lamina of the aortic wall can lead to aneurysm formation [3, 4]. Risk factors for AAA formation include hypertension, smoking, age greater than 60 years, and male sex [3–5]. If untreated, continued dilation can potentially lead to rupture, which presents as a pulsatile abdominal mass and tearing pain radiating to the back [5].

Elective surgical repair of AAA significantly decreases mortality. Open surgical repair (OSR) or endovascular placement of aortic stent graft (EVAR) are the two surgical methods available for AAA repair. EVAR is the preferred modality as it is minimally invasive and has lower postoperative complication rates and mortality [5]. Elective AAA repair is recommended for aneurysms larger than 5.5 cm in men, larger than 5 cm in women, symptomatic aneurysms, or aneurysms expanding by more than 1 centimeter per year [5].

Patients with AAA and CAD may potentially require both of these procedures. Previously shown to reduce secondary aneurysm rupture, very ill patients may maximize mortality benefit from a combined CABG and AAA repair instead of a staged approach [6]. Here, we present a rare case of a 71-year-old male presenting with an expanding AAA and that underwent a combined CABG and AAA repair approach.

## Case description

**Figure 1:**
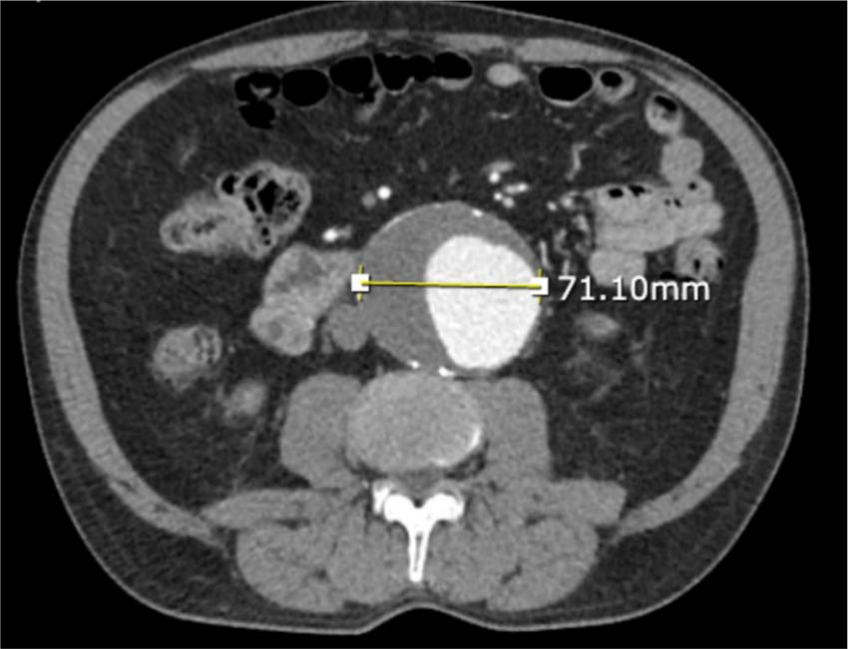
CT angiogram of abdominal aorta and iliofemoral artery axial view.

**Figure 2:**
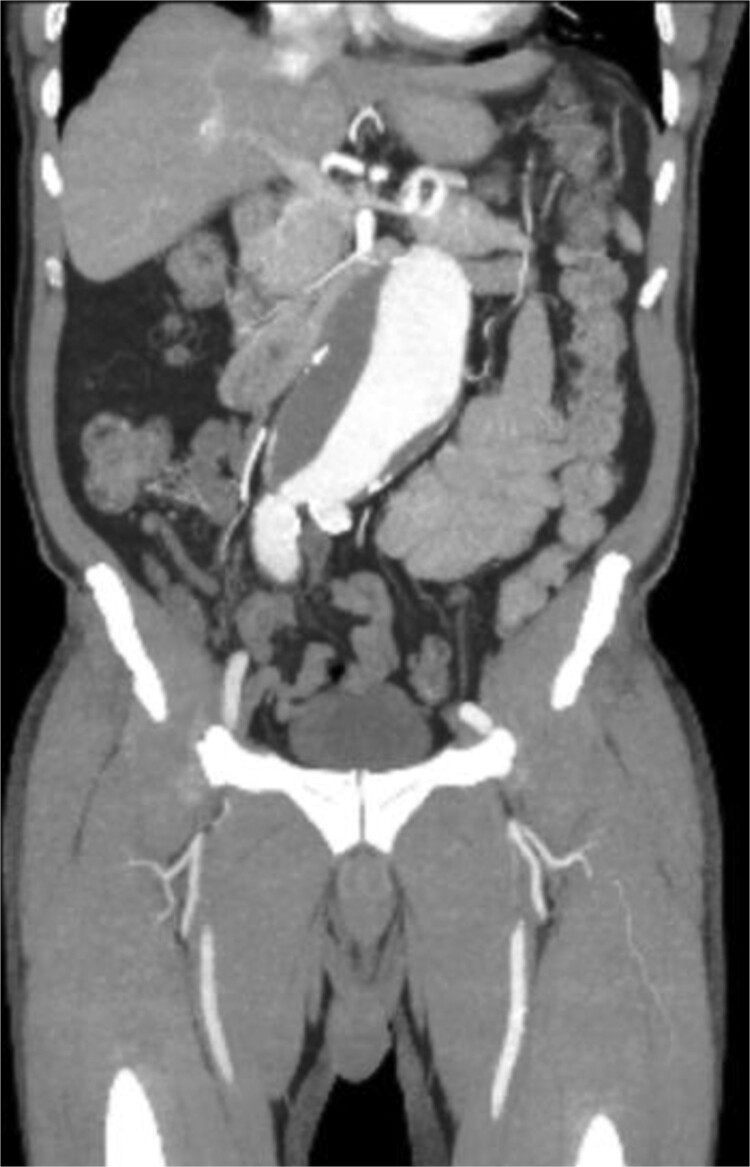
CT angiogram of abdominal aorta and iliofemoral artery coronal view.

A 71-year-old male with a past medical history of hypertension, dyslipidemia, and renal insufficiency presented to the emergency department with headache and dizziness that began after performing manual labor. The only abnormality with initial vital signs was an elevated blood pressure. Physical exam revealed a palpable pulsatile mass in the epigastrium. An electrocardiogram showed normal sinus rhythm with signs of previous inferior and anterolateral infarcts. Troponin levels were within normal limits. Further workup with an echocardiogram demonstrated left ventricular hypertrophy and ejection fraction (EF) of less than 50%. Due to the pulsatile abdominal mass, a computed tomography (CT) of the abdomen without contrast, and subsequent CT angiogram of the abdomen and pelvis, was completed that demonstrated a 7.1-cm infrarenal AAA with a large mural thrombus causing luminal narrowing (Figs 1 and 2).

Vascular surgery was consulted at this time for AAA evaluation. To better understand the patient’s cardiovascular status, a nuclear stress test was conducted showing two prior myocardial infarctions and EF of 30%. These findings prompted left heart catheterization, which revealed severe cardiomegaly, near-total occlusion of both the left anterior descending (LAD) and right coronary arteries (RCA), subtotal lesion in the left circumflex artery and EF of 25%.

Due to the coronary arterial occlusions, cardiothoracic surgery evaluated the patient. Their evaluation determined that the patient required urgent CABG for myocardial revascularization prior to AAA repair. Given the patient’s comorbidities, the patient was deemed to be a high-risk candidate for multiple surgeries. Therefore, a joint decision was made to perform a combined surgery where the CABG would allow for coronary revascularization followed immediately by an open AAA repair.

After the patient was brought to the operating room, CABG was performed first. The left internal mammary artery was grafted to the LAD, and the right saphenous vein was used for reverse grafting to the obtuse marginal branch and posterior descending artery. The cardiac portion of the surgery was completed successfully. Following closure of the thorax, the vascular surgery team proceeded with the open AAA repair. The aneurysm, which extended from the infrarenal aorta into the right common iliac artery, was ligated as far right as possible and aortic neck exposed appropriately. Aneurysm incision was continued until enough viable aortic wall was visualized for subsequent anastomosis of the 18-mm bifurcated aortic graft to the aorta and iliac arteries. The abdomen was closed without complication and the patient tolerated the entire surgical procedure well.

Postoperatively, the patient was transferred to the cardiothoracic intensive care unit. Immediately postoperatively, he required vasopressors and continuous veno-venous hemofiltration. However, vasopressor requirements decreased and renal function improved over subsequent days. He was later transferred to the cardiac step-down unit for ongoing cardiac rehabilitation and was discharged on postoperative day 10. Follow-up evaluations at one and three months postoperatively showed significant recovery.

## Discussion

This case report highlights the successful management of a 71-year-old male patient by combined CABG and open AAA repair. Currently, the standard of care is to perform a CABG followed by AAA repair two to six months later [7]. However, conducting both procedures concomitantly, though rare, has potential benefits for patients with severe comorbid conditions. Such advantages include reducing surgical and recovery time, decreased anesthesia exposure, and elimination of possible AAA rupture post-CABG [8–10]. However, preoperative evaluation is crucial and current guidelines recommend careful selection of patients; not all patients with concurrent CAD and AAA will be suitable candidates for such an approach [4, 5, 7, 11]. Limited literature describes an ideal patient being one with minimal comorbidities, a large stable aneurysm and preserved ejection fraction [7]. However, the patient presented had significant comorbidities and compromised ejection fraction. Our case illustrates that high-risk candidates that may not meet ideal criteria can potentially benefit from combining these procedures. Furthermore, surgeries must be considered on an individual patient basis with clinical judgment.

Ultimately, further research is warranted to establish more definitive patient selection criteria and indications for a combined procedure. Future comparative studies of staged versus combined procedures will provide clarity and insight into management strategies for more complex patients.
